# Surface plasmon-cavity hybrid state and its graphene modulation at THz frequencies

**DOI:** 10.1515/nanoph-2023-0643

**Published:** 2024-01-08

**Authors:** Yifei Zhang, Baoqing Zhang, Zhaolin Li, Mingming Feng, Haotian Ling, Xijian Zhang, Xiaomu Wang, Qingpu Wang, Aimin Song, Hou-Tong Chen

**Affiliations:** Shandong Technology Center of Nanodevices and Integration, School of Integrated Circuits, Shandong University, Jinan, 250100, China; National Laboratory of Solid State Microstructures, School of Electronic Science and Engineering, Nanjing University, Nanjing, 210023, China; Department of Electrical and Electronic Engineering, University of Manchester, Manchester, M13 9PL, UK; Center for Integrated Nanotechnologies, Los Alamos National Laboratory, Los Alamos, NM, 87545, USA

**Keywords:** hybrid surface state, Fabry–Pérot cavity, surface plasmon, graphene, terahertz

## Abstract

Fabry–Pérot (F–P) cavity and metal hole array are classic photonic devices. Integrating F–P cavity with holey metal typically enhances interfacial reflection and dampens wave transmission. In this work, a hybrid bound surface state is found within rectangular metal holes on a silicon substrate by merging an extraordinary optical transmission (EOT) mode and a high-order F–P cavity mode both spatially and spectrally. Transmission, Q-factor, and bandwidth can be enhanced significantly with respect to the classical EOT and F–P interference by simply sweeping the cavity length. This state can provide EOT properties and ten times broader EOT bandwidth well below the effective plasma frequency of the periodic metal holes, where the metal holes typically show evanescent properties and do not support EOT in theory. Furthermore, a large modulation range of 25 % and 39 % is demonstrated with various graphene patterns for the transmittance of this hybrid state at 500 and 582 GHz, respectively.

## 1 Introduction

Fabry–Pérot (F–P) cavities and subwavelength periodic metal hole arrays are canonical photonic structures and have stimulated renewed research hotspots, such as extraordinary optical transmission (EOT), vertical-cavity surface-emitting laser, and quantum optics [1]–[5]. Integrating a dielectric F–P cavity with metal hole arrays can enhance the interfacial reflection and thus the quality (Q) factor at the cost of reduced wave transmission below the plasma frequency of the metal hole arrays, i.e., the asymptotic limit in the spectrum for the dispersion relation [6]. Typically, the Q-factor of such F–P cavities decreases as the cavity order increases and shows the maximum at the first-order cavity mode [6], [7].

At the plasma frequency, metal holes boost wave transmission collectively with a transmittance larger than the hole area ratio, which is referred to as EOT [8]. Two surface plasmon (SP) modes on the top and bottom metal surfaces weakly couple to each other through the holes to promote wave transmission [9]. Surface plasmons are electromagnetic surface excitations trapped at a metallic surface through their interaction with the metal free electrons, which were originally reported at optical frequencies [10]. Pendry et al. revealed terahertz (THz) EOTs on the perforated metal films by an effective permittivity of the same plasma form as the optical SPs [11]. Below the effective plasma frequency, the metal surface shows little diffraction and small wave transmission [8]–[12].

In this work, a SP-cavity bounded surface state is discovered on a metal film with simple square holes and a silicon substrate in the THz regime, which can provide enhanced transmission, Q-factor, and bandwidth by tailoring the F–P cavity length. This hybrid state comprising one effective SP mode and one high-order F–P cavity mode exists at or below the plasma frequency of the metal hole array. At the plasma frequency, its Q-factor may increase several times as the F–P mode order increases, and the corresponding transmission is also enhanced over the classic EOT. Below the plasma frequency, the transmission may remain stronger than the classic EOT and show a 10-times broader passband. Note that no F–P cavity resonance can be found above the plasma frequency on the structure, except the first-order one. Finally, active modulation of this state has been investigated by using various graphene patterns and different biasing methods. The absolute modulation range can be as large as 39 %, which to our knowledge reaches the best for graphene-based active EOTs.

## 2 Theory and analysis

**Figure 1:**
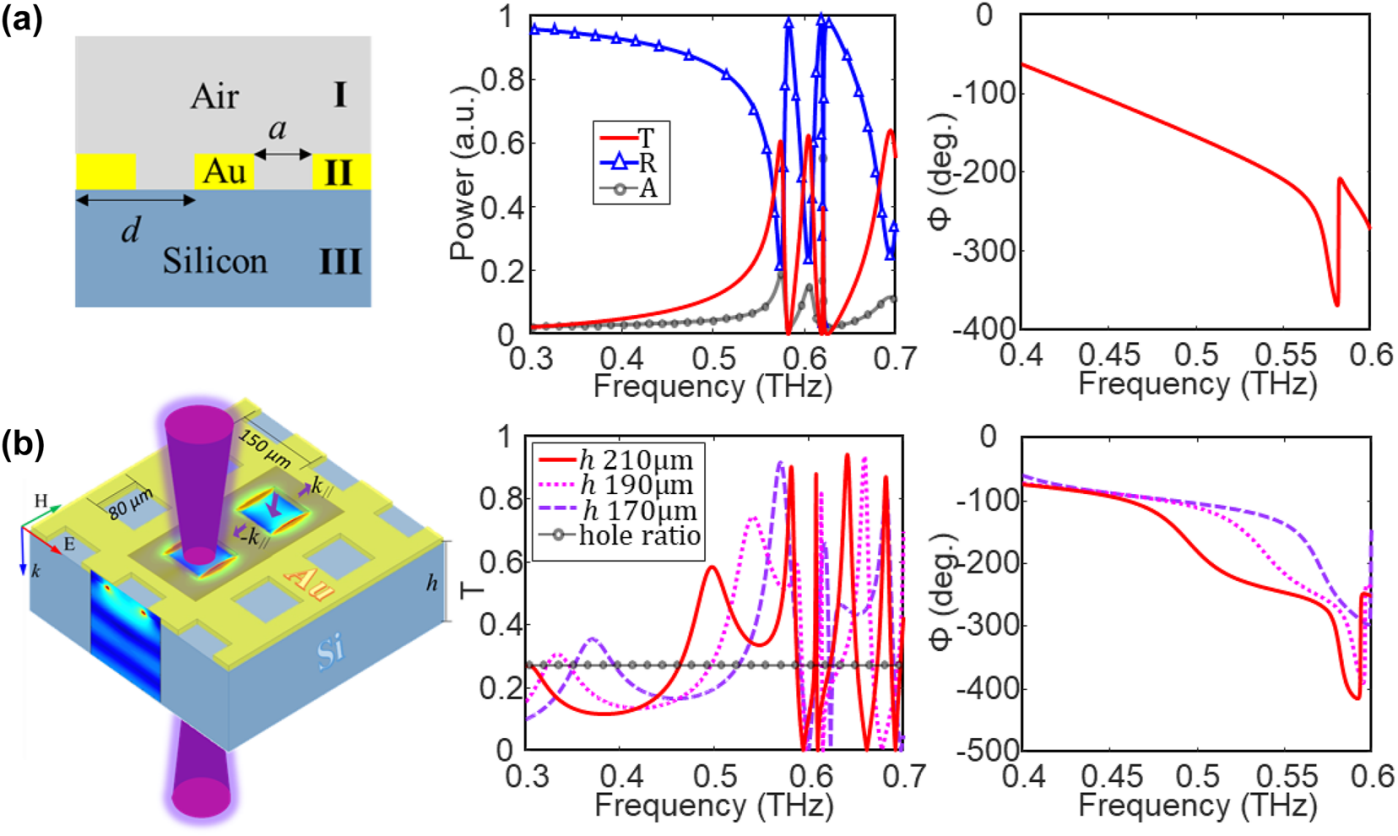
Periodic metal hole arrays on silicon substrates with infinite and finite thickness. (a) Canonical model of a metal hole array sandwiched by semi-infinite air and silicon layers. A sharp phase variation can be found at the plasma frequency *f*_*pl0*_ = 575 GHz, which corresponds to the first-order EOT. The transmittance is 0.61 at 575 GHz, and the Q-factor is 30. (b) 3-D model of the hybrid state on square metal holes and finite silicon substrate. It consists of one spoof SP mode and one F–P cavity mode. The transmittance of the hybrid state is stronger than the ideal EOT at and below *f*_eff_ (the resonant frequency of the first-order EOT mode). The Q-factor is enhanced to 76 at *h* = 210 μm.

Figure 1(a) illustrates a simple metal hole array sandwiched by air and silicon, which has a dispersive dielectric function *ɛ*_*m*_(*ω*). The periodic rectangular holes have a length of *a* = 80 μm, a lattice period of *d* = 150 μm, and a height of 0.3 μm. Light incidents normally from the air and radiates to the silicon. The simulated transmittance, reflectance, and absorption are also shown in Figure 1(a). Two EOT peaks with Fano line-shape are found at 575 and 605 GHz, respectively, and a transmission zero lies between these two peaks, revealing a classic EOT phenomenon. The transmittance is as strong as 0.61 at 575 GHz, which is two times larger than the hole area ratio. The wavelength of the first order SP mode is estimated as $\lambda =2\sqrt{\varepsilon_{r}}a$ [9], and the first transmission zero is found at $\lambda =\sqrt{\varepsilon_{r}}d$ , where *ɛ*_*r*_ is the effective permittivity of the substrate [8]. The half-power bandwidth of the first-order EOT at *f*_eff_ = 575 GHz is approximately 3.3 %, and the corresponding Q-factor is 30. Below *f*_eff_, the subwavelength holes show little transmission and strong reflection.

**Figure 2:**
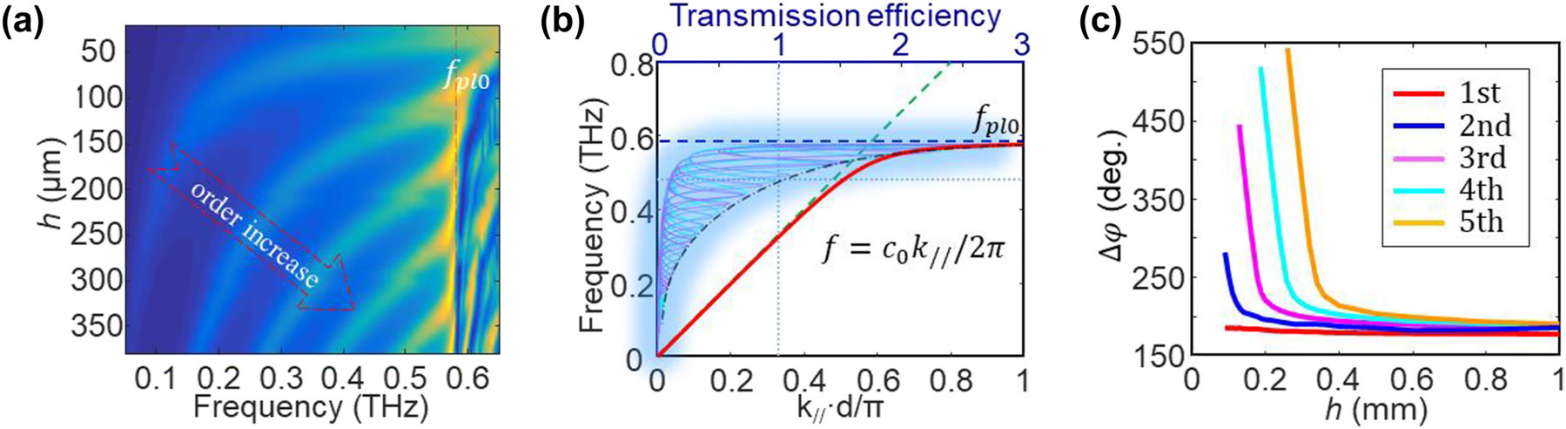
Transmittance magnitude and phase of the periodic metal holes on silicon substrates with various thickness. (a) Transmittance variation with respect to silicon thickness and frequency. The hybrid state only exists with high-order F–P cavity modes, and its Q-factor increases as the order of the F–P cavity increases. (b) Dispersion relation of surface plasmon on the metal hole array (the red line). The green dashed line depicts a linear curve for the light. The shadowed curves are the transmission efficiencies of the hybrid states with various F–P resonant orders and substrate thickness. They increase from unity to 2.75 as the SP line deviates from the light line. (c) Δ*φ* induced by the SP mode at various F–P cavity orders. It exponentially increases due to the enhanced slow-wave effect as the F–P resonance approaches *f*_eff_.

Next, we consider a complex model with finite silicon layer in Figure 1(b). The simulated EOT slightly blue shifts to 583 GHz due to the finite silicon thickness. A series of enhanced transmission peaks are found below *f*_eff_, whose wavelengths are related to the F–P resonances by

(1) $$2\sqrt{\varepsilon_{r}}h+\frac{\Delta \varphi }{2\pi }\lambda =n\lambda ,$$

where *λ* is the wavelength in free space, Δ*φ* is the phase shift due to the perforated metal film, and *n* is an integer. The transmittance mapping for the hybrid state with different cavity orders is illustrated in Figure 2(a). For each order F–P cavity mode, the Q-factor of the hybrid mode decreases as *h* increases. The F–P cavity modes disappear right at *f*_eff_ and do not exist above *f*_eff_ except the first-order one, showing an interesting cut-off phenomenon. The hybrid state can provide higher transmission efficiency than the classic SP EOT even below the plasmonic frequency *f*_eff_, as shown in Figure 2(a), and can push EOT phenomenon down to 470 GHz, as shown in Figure 2(b), which corresponds to a red-shift factor of as large as 20 %. In addition, a broadband EOT composing two transmission peaks can be tailored with a bandwidth of 13 % by varying *h* (190 µm).

Typically, a metal film with square hole array and a metal surface covering a layer of dielectric can bind a bound surface state [11]. The former plays a key role in the classic SP EOTs, whose dispersion relationship is

(2) $$k_{//}^{2}c_{0}^{2}={\left(2\pi f\right)}^{2}+\frac{1}{f_{\text{eff}}^{2}-f^{2}}\frac{256a^{4}f^{4}}{\pi^{2}d^{4}},$$

where *k*_//_ is the wavenumber of SPs. As depicted in Figure 2(b), the calculated dispersion relation (the red line) gradually deviates from the light line (the green line) and approaches the effective plasma frequency *f*_eff_, revealing enhanced slow-wave effect [9]. The holey metal induces extra phase term Δ*φ* for the F–P cavity, which is depicted as

(3) $$\frac{\Delta \varphi }{\Delta l}=\frac{2\pi }{\lambda }=k_{//},$$

where Δ*l* is the length for Δ ***φ*** on the metal holes. According to Equation (1), Δ ***φ*** can be derived from the frequencies of the hybrid states, which is plotted in Figure 2(c). At the frequencies far below ***f***_***eff***_, Δ ***φ*** roughly equals 180°, which corresponds to the reflection of the metal film. As the frequency approaches ***f***_***eff***_, Δ ***φ*** ramps significantly due to the enhanced slow-wave effect, and so does ***k***_//_, see Figure 2(c), and the transmittance increases over the classic EOT, as shown in Figure 1(b). An anti-crossing effect is observed between the low-order hybrid mode and the spoof SPP mode in Figure 2(a). As the thickness increases, the low-order F–P mode cannot support the low-order hybrid mode so that the hybrid mode suddenly changes to the spoof SPP mode. Correspondingly, the Q-factor changes sharply, as shown in the Supplementary Information.

### 2.1 Experiment

**Figure 3:**
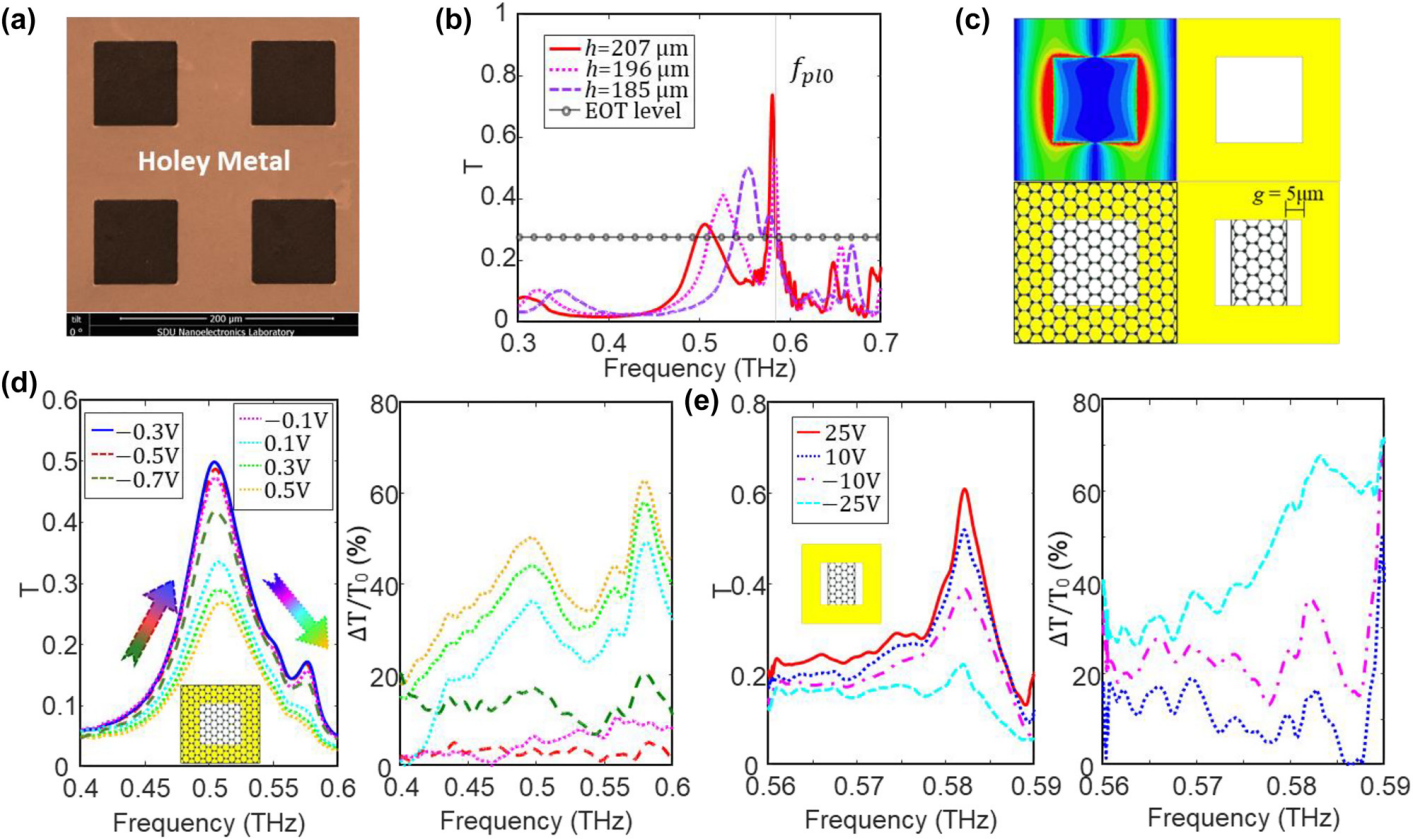
Periodic metal holes with graphene modulation. (a) SEM image of a fabricated metal hole array on silicon substrate. (b) Characterized transmittances with various silicon thickness *h*. The transmittance of the hybrid mode is larger than the EOT below the effective plasma frequency (*f*_eff_ = 583 GHz). (c) Two graphene patterns for the active modulation of rectangular metal holes. (d) Active modulation and modulation depth of the hybrid mode at 500 GHz with a graphene film and PSSNa on a 196-μm thick silicon substrate. The graphene is gated by using PSSNa. (e) Active modulation and modulation depth of the hybrid mode at 582 GHz with patterned graphene strip on a 207-μm thick silicon substrate. The graphene is gated by using silicon substrate.

Figure 3(a) illustrates the fabricated metal holes with a size of 80 μm and a lattice period of 150 μm on silicon substrates with 100-nm SiO_2_. The metal thickness is 0.3 μm, and the silicon thickness *h* sweeps from 185 to 207 μm. Toptica THz frequency-domain spectroscopy is utilized to characterize the transmittances, which is shown in Figure 3(b). The hybrid bound state addresses an enhanced transmittance of 0.73 at 582 GHz for (*h* = 207 μm), and the corresponding Q-factor is as high as 80, i.e., around three times higher than the classic EOT. No high-order F–P cavity modes can be found above *f*_eff_. In addition, a broadband EOT phenomenon with two transmission peaks and no transmission zeros between the peaks can be found at *h* = 185 μm, revealing a half-power bandwidth of 10 %, i.e., three times larger than the classic SP EOT.

## 3 Graphene modulation

Active modulation of this hybrid mode is investigated with various graphene patterns and different biasing methods, as shown in Figure 3(c). We choose two specific frequencies, i.e., 500 and 582 GHz, for the F–P mode dominant case and the SP mode dominant case, respectively. Their experimental data are shown in Figure 3(d) and (e).

After the fabrication of the metal holes on a 196-μm silicon substrate, a chemical vapor deposition (CVD) graphene was transferred onto the film, and then solution-processed poly (styrenesulfonic acid sodium salt) (PSSNa) was spin-coated on the graphene with a thickness of 70 μm for top-gating. Due to the graphene attenuation, the transmittances of the hybrid modes reduce to 0.5 and 0.17 at 500 and 580 GHz, respectively, at the Dirac point (−0.3 V). This phenomenon reveals that the SP mode is more dominant near the effective plasma frequency and becomes less dominant as the frequency decreases. Furthermore, adding a thin dielectric layer on top of the metal holes can further push EOTs to 366 GHz, as shown in the Supplementary Information, which approximately corresponds to a red-shift factor of 38 % with respect to the SP EOT. The spectral transmittances at various biases and the corresponding modulation depth are illustrated in Figure 3(d). The transmittance at 500 GHz enlarges from 0.42 to 0.5 under a bias sweeping from −0.7 to −0.3 V and reduces from 0.5 to 0.25 under a bias sweeping from −0.3 to 0.5 V. Meanwhile, the transmittance at 578 GHz is also modulated from 0.17 to 0.07. The corresponding modulation depth is larger than 50 % and 60 % at 500 and 580 GHz, respectively.

To enlarge the absolute modulation range and suppress the graphene attenuation near *f*_eff_, graphene strips are designed for the SP mode dominant case with a distance of *g* = 5 μm from the hole edge, which avoids the concentrated electric fields, see Figure 3(c). Here, silicon substrate is utilized as the bottom electrode for gating graphene, and the Dirac point is around 25 V. In this case, the transmittance at 582 GHz is slightly suppressed to 0.61, which can be modulated to 0.22 by sweeping the bias voltage from 25 to −25 V, as depicted in Figure 3(e). The corresponding modulation depth is around 70 % at 582 GHz. Here, it is worthy to mention that the modulation range of our device to our knowledge is the state-of-art for active EOTs with graphene, and the applied bias with PSSNa is among the lowest ones for graphene tuned metamaterials [13]–[17], as shown in the Supplementary Information Table I.

## 4 Conclusions

A novel SP-cavity hybrid state was found on rectangular metal holes on a silicon substrate at THz frequencies, which may provide large Q-factor, high transmittance, and broad bandwidth below the effective plasmonic frequency of the classic EOT with careful design. A large modulation range of 25 % and 39 % was demonstrated for this state at 500 and 582 GHz with different graphene patterns and biasing methods, respectively. Recently, THz technologies are rising for an increasingly wide variety of applications [18]–[22], such as biosensing and medical diagnosis, ultrahigh-speed communication, quantum optics, etc. This hybrid state provides a novel approach with more subwavelength holes for sensing molecules and chemicals as well as for studying THz quantum devices. Furthermore, its tailorable broadband, high transmission, high Q-factor, and large modulation range may enable stand-alone filters, polarizers, and lenses for the advanced THz communication and imaging applications. Finally, the reported active modulation methods make these applications compatible with typical silicon complementary metal–oxide–semiconductor (CMOS) bias voltages.

## 5 Methods

### 5.1 Sample fabrication

The metal films were fabricated with standard ultraviolet photolithography (AZ6130 photoresist), E-beam evaporation (HHV Auto500), and lift-off process. To achieve various substrate thickness, the silicon substrates were etched by using inductively coupled plasma (ICP) etching system (Oxford PlasmaPro 100 Cobra). The etch rate is 2.8 μm/min under a RF power of 30 W, an ICP power of 825 W, and a mixed gas of O_2_, SF_6_ and Ar (2 sccm, 26 sccm and 20 sccm).

### 5.2 Ion gel preparation

PSSNa, D-sorbitol, glycerol and DI water (with a weight ratio of 40, 10, 10, and 40 %) were mixed with magnetic stirring. After stirring for 2 h at room temperature, the ion gel was spin-coated onto the metallic film with a spinning rate of 1000 rpm for 1 min.

### 5.3 Characterization

The transmittances were characterized using Toptica TeraScan 1550 THz frequency-domain spectroscopy (FDS) at room temperature, which are normalized to the water vapor absorption lines with stable humidity. The incident terahertz wave was generated by using two continuous wave lasers with differential frequency method and was focused with a beam radius of around 2 mm in a four-mirror reflection system. The spectral resolution is as small as 10 MHz.

### 5.4 Simulation

The 3-D models of the EOT structures are simulated with Master and Slave boundaries, i.e., a kind of periodic boundary, in Ansys High Frequency Structural Simulator (HFSS).

## Supplementary Material

Supplementary Material Details
