# Supplementary material for: Surface plasmon-cavity hybrid state and its graphene modulation at THz frequencies
Source: Nanophotonics. 2024 Jan 8;13(12):2207–12. doi: 10.1515/nanoph-2023-0643 (PMC11501170; doi:10.1515/nanoph-2023-0643)
Supplement: Supplementary file 1 — Supplementary Material Details [file j_nanoph-2023-0643_suppl_001.docx]

**Supporting Information:**

# Surface Plasmon-Cavity Hybrid State and Its Graphene Modulation at THz frequencies

Yifei Zhang^1#*^, Baoqing Zhang^1#^, Zhaolin Li^1^, Mingming Feng^1^, Haotian Ling^1^, Xijian Zhang^1^, Xiaomu Wang^2^, Qingpu Wang^1^, Aimin Song^1,3 *^, and Hou-Tong Chen^4 *^

^1^*Shandong Technology Center of Nanodevices and Integration, School of Integrated Circuits, Shandong University, Jinan, 250100, China*

^2^*National Laboratory of Solid State Microstructures, School of Electronic Science and Engineering, Nanjing University, Nanjing, 210023, China*

^3^*Department of Electrical and Electronic Engineering, University of Manchester, Manchester, M13 9PL, United Kingdom*

^4^*Center for Integrated Nanotechnologies, Los Alamos National Laboratory, Los Alamos, New Mexico, 87545,United States*

^*^Author to whom correspondence should be addressed. E-mail: yifeizhang@sdu.edu.cn

^*^Author to whom correspondence should be addressed. E-mail: [a.song@manchester.ac.uk](mailto:a.song@manchester.ac.uk)

^*^Author to whom correspondence should be addressed. E-mail: chenht@lanl.gov

^#^Authors contribute equally to this work

### Electric field distributions

| 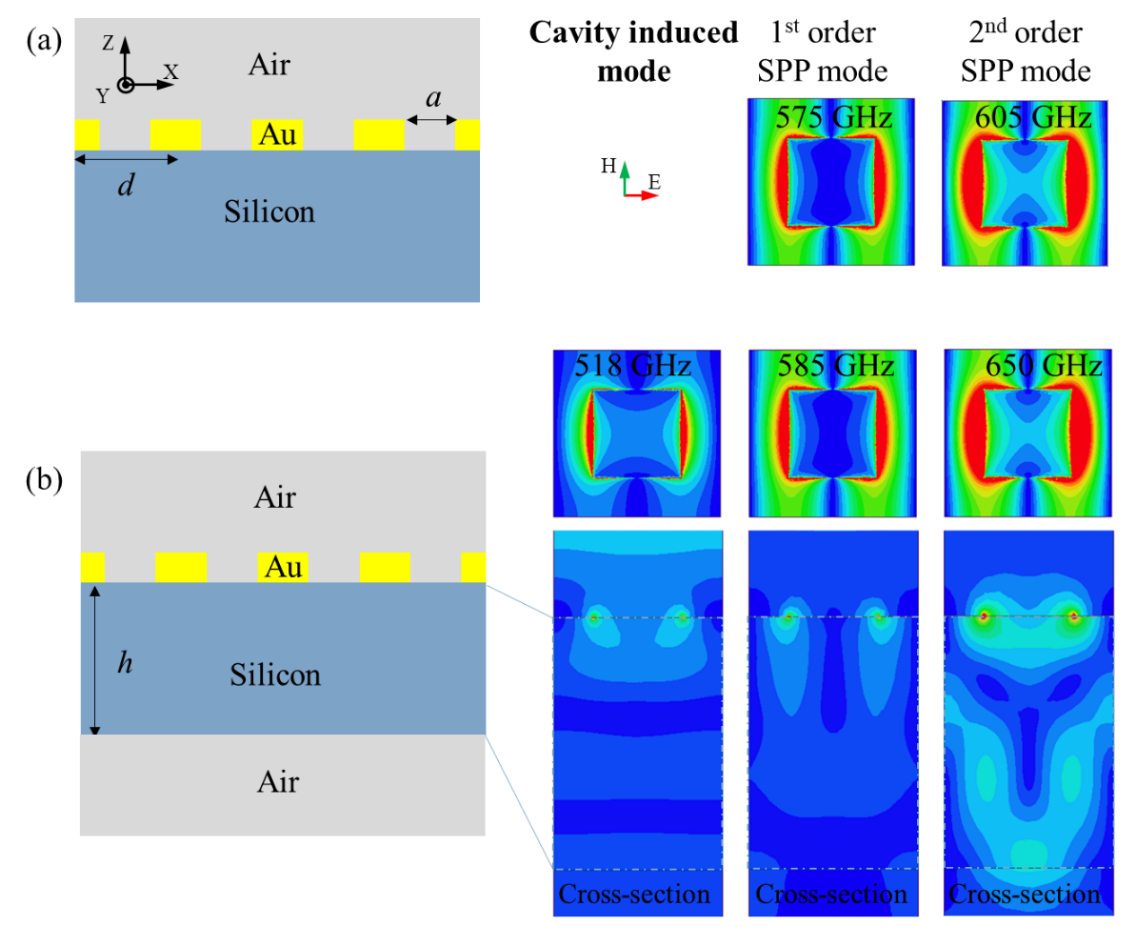 |
| --- |
| Figure S1. Electric field distributions of a metallic film perforated with square hole array on silicon substrate with (a) infinite and (b) finite thickness at EOT frequencies ($a=80 \mu m, d=150 \mu m,h=200 \mu m$). The first and second order SPP modes blue shifts to 585 and 650 GHz as the substrate thickness gets finite. |

Figure S1(a) illustrates a canonical model of a holey metal film sandwiched by two semi-infinite media, i.e., air and silicon. The width, height, and lattice period of the square holes are 80, 0.3, and 150 μm, respectively. Terahertz wave normally incidents along the Z-axis from the air side with its electric fields polarized in the X-direction, and excites two surface plasmon (SP) modes for extraordinary optical transmissions (EOTs). The electric field distributions of the two classic EOTs at 575 and 605 GHz are illustrated in Figure S1(a), revealing standing-wave properties and a diffraction angle of 90º. Figure S1(b) depicts a practical model with a finite substrate thickness of 200 μm. The subwavelength thickness blues shifts the first and second order SPP modes to 585 and 650 GHz, whose electric field distributions remain the same. A novel hybrid bound state consisting of one [Fabry–Pérot](https://en.wikipedia.org/wiki/Fabry%E2%80%93P%C3%A9rot_interferometer) (F-P) cavity and one SP mode can be found at 518 GHz, which shows EOT properties well below the plasma frequency. It can be seen that the hybrid state has similar field distributions as the classic EOT and shows better field confinement in the X- and Y-directions. However, the mode profiles are different in the Z-direction.

### F-P interference effect

Typically, a dielectric reflector induces zero phase shift for the reflected waves, and a metallic reflector induces 180º phase shift for the reflected waves. In our F-P cavity with a dielectric reflector and a metallic reflector, the resonant wavelength is depicted as

S-(1)

S-(1)

$$\frac{(2n-1)\lambda}{2}=2\sqrt{\varepsilon_{r}}h,$$

| 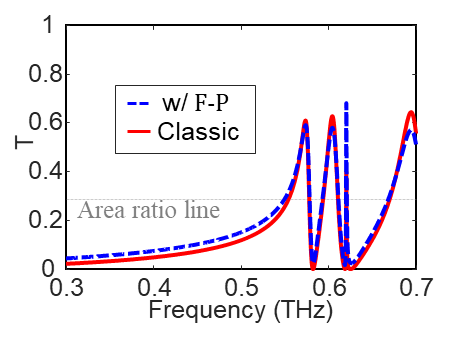 |
| --- |
| Figure S2. Transmittances with and without the effect of F-P interference. The red line is the transmittance of the classic model in Figure S1(a) without F-P interference, and the blue line is the calculated transmittance considering the F-P interference. The F-P cavity slightly enhances the transmittance below the first order plasma frequency. |

where *h* is the substrate thickness, *n* is an integer, and $\varepsilon_{r}$ is the effective permittivity of the substrate. The reflectance of the holey metal film ($R_{1}$) can be found as the blue line of Figure 1(a) in the main manuscript, and the reflectance of the silicon-air interface is

S-(2)

$$R_{2}=\left( \frac{n_{1}-n_{2}}{n_{1}+n_{2}} \right)^{2}=\left( \frac{\sqrt{11.9}-1}{\sqrt{11.9}+1} \right)^{2}=0.303.$$

Considering the F-P interference, the total transmittance of a holey metal film on a 200-μm thick silicon substrate can be calculated using the following equation

$$T_{0}=\frac{(1-R_{1})(1-R_{2})}{1-R_{1}R_{2}}.$$

The calculated data are plotted as the blue dashed line in Figure S2, which is slightly larger than the transmittance without F-P interference below the first order plasma frequency. However, the transmittance with the effect of F-P interference is much weaker than the cavity-induced EOT below the first order plasma frequency. Taking 519 GHz for instance, the transmittance with F-P interference is merely 0.18, and the transmittance of the hybrid state is as high as 0.4.

S-(3)

### SP properties of the enhanced transmission modes

| 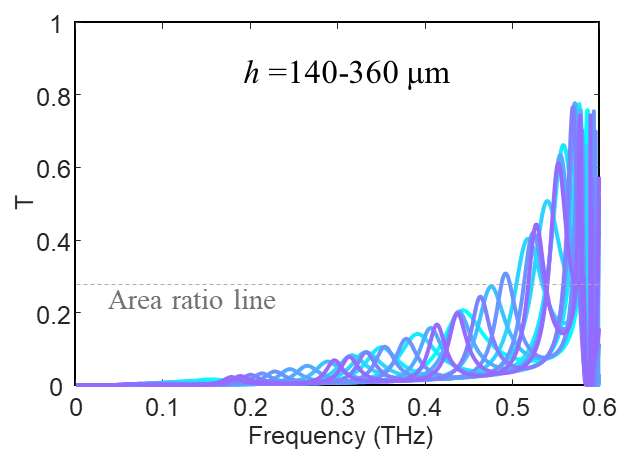 | | 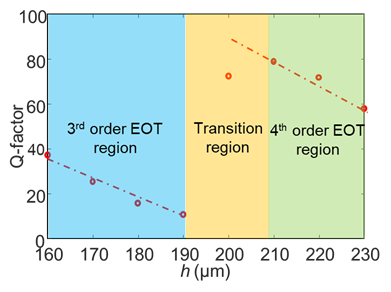 | |
| --- | --- | --- | --- |
| (a) | (b) | | |
| 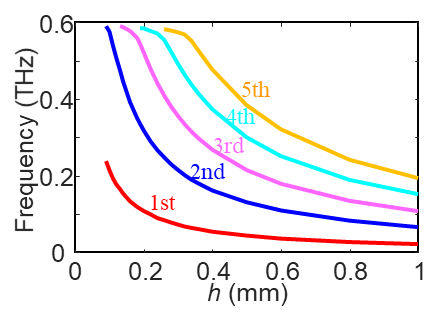 | | | 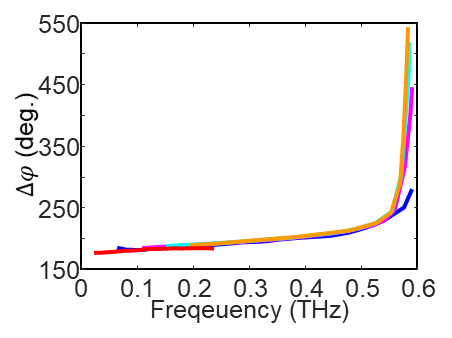 |
| (c) | | | (d) |
| Figure S3. (a) Enhanced transmission modes with various orders on a silicon substrate with different thicknesses. (b) Dependence of the Q-factor on h. (c) Resonant frequency of these modes as a function of substrate thickness. As the substrate gets thinner, the resonant frequency is truncated at the first order plasma frequency. (d) Phase item induced by SPs on the holey metallic film as a function of frequency. $\Delta\varphi$ ramps fast near the plasma frequency. | | | |

Figure S3(a) depicts the enhanced transmission modes with various F-P resonant orders on a silicon substrate with different thickness. The mode transmittance enlarges as the frequency approaches the first order SP EOT mode due to the enhancement of the SPs. The modes with a transmittance larger than the hole area ratio lie above 471 GHz, which can be classified as EOT by definition. In this case, EOT phenomenon has been pushed deep into the sub-wavelength region by a factor of as large as 20%. These EOTs are induced by a novel hybrid bound surface state comprising the F-P resonant mode and the SPs on the metal film, see Figure S1(b). The Q-factor of this hybrid state as a function of substrate thickness *h* is illustrated in Figure S3(b). For each order F-P cavity mode, the Q-factor of the hybrid mode decreases as h increases. The resonant frequency of the hybrid state gradually stops at the plasma frequency due to the enhanced slow-wave effect of the SPs. This phenomenon can be explained by using the phase term $\Delta\varphi$ in Equation (1), whose variation at different frequencies is depicted in Figure S3(d). $\Delta\varphi$ induced by the slow-wave effect ramps quickly near the first order plasma frequency. As the frequency of the enhanced transmission mode gets far away from the first order plasma frequency, the holey metal induces a phase shift of 180°as the classic metal reflector.

### Transmission enhanced with ion gel

| 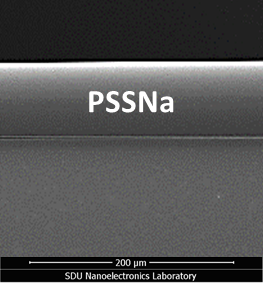 | 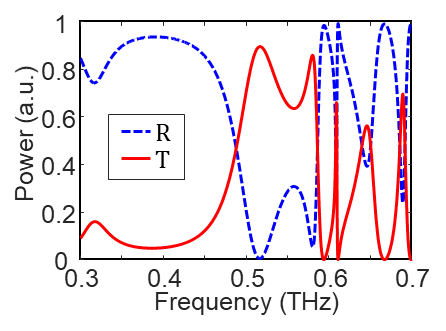 |
| --- | --- |
| (a) | (b) |
| 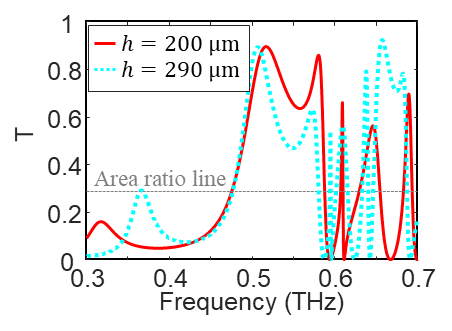 | 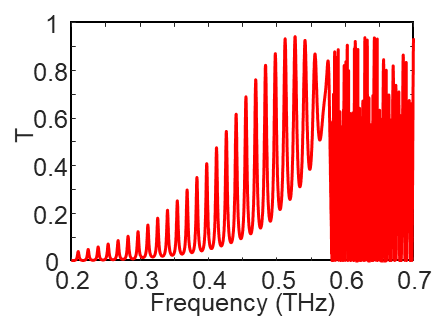 |
| (c) | (d) |
| Figure S4. (a) SEM image of the spin-coated ion gel with a thickness of 70 μm. Transmittance enhanced by the spin-coated ion gel with a substrate thickness *h* of (b) 200 μm and (c) 290 μm. (d) periodic peaks below the first order plasma frequency (*h* = 3 mm). | |

Garcia-Vidal et al. reported filling the hole with a dielectric with its permittivity larger than unity enhances the magnitude of EOTs.^S1^ We found the same phenomenon for the hybrid state in our simulation with ion gel. The spin-coated ion gel layer has a thickness of 70 μm, as illustrated in Figure S4(a), and a relative permittivity of around 3,^S2^ which significantly enhances the hybrid state and the first order SP EOT simultaneously in Figure S4(b). The former increases to 0.896 at 510 GHz, and the latter increases to 0.863 at 580 GHz. The corresponding transmission efficiencies are as high as 3.15 and 3.03, respectively. A wide band from 475 to 588 GHz shows a transmission efficiency larger than unity, revealing a broadband EOT phenomenon with a bandwidth of 22%. Furthermore, adding a dielectric layer on the top of the holey metal film can further push the EOTs deep in the sub-wavelength region, such as 366 GHz in Figure S4(c), which corresponds to a red-shift factor of 38%. In this case, thick substrate induces periodic transmission peaks below the first order plasma frequency, as illustrated in Figure S4(d).

### Active modulation simulation

| 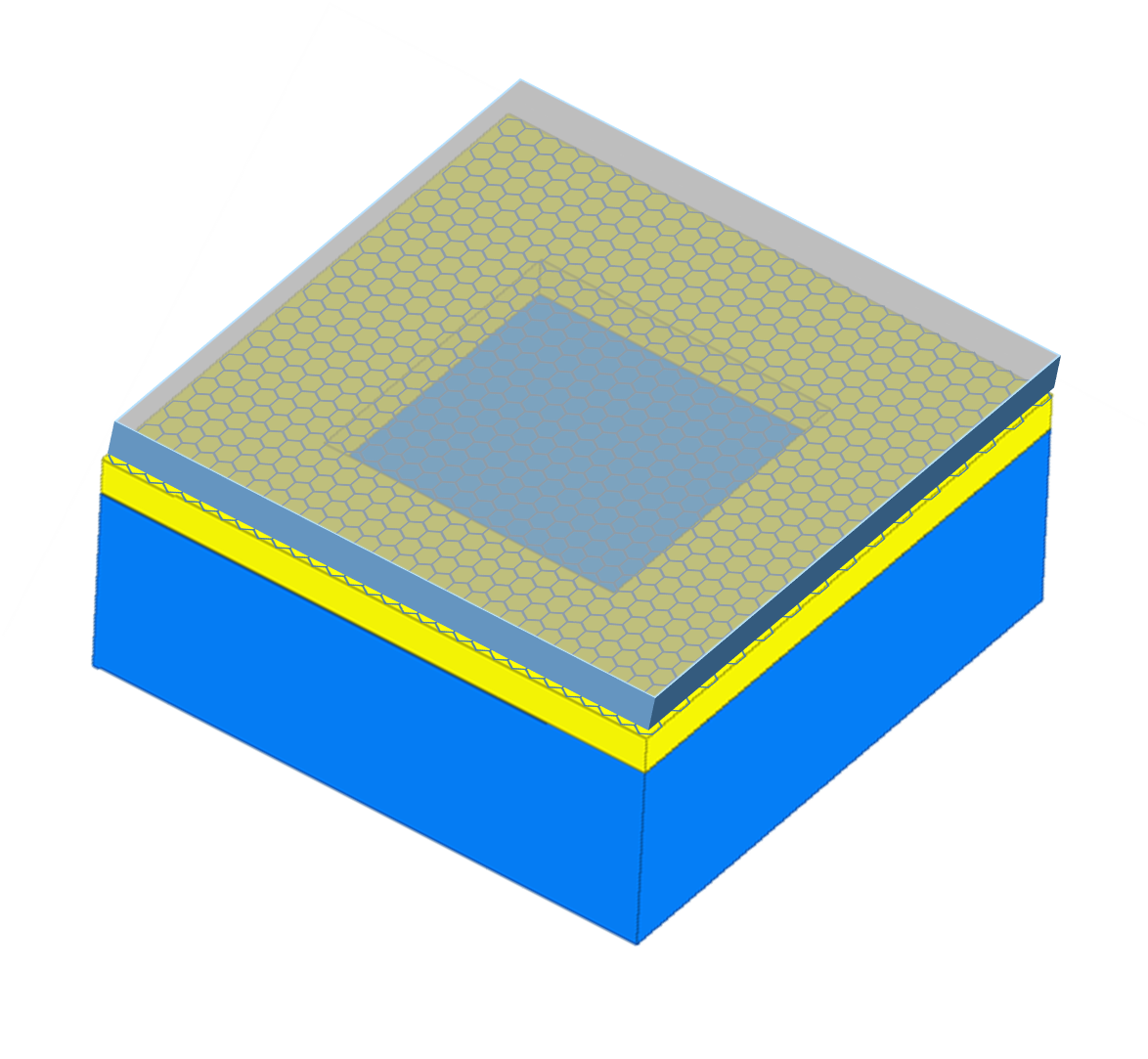 | 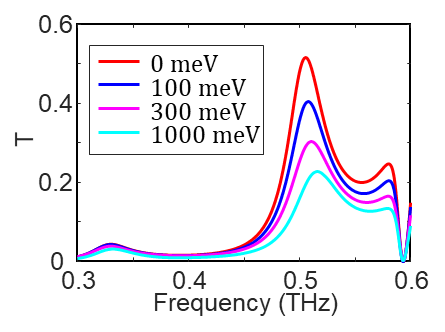 |
| --- | --- |
| (a) | (b) |
| 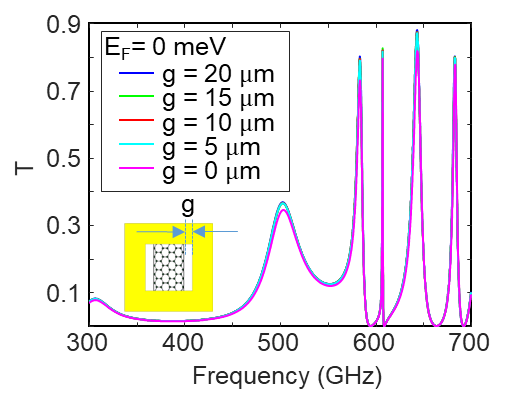 | 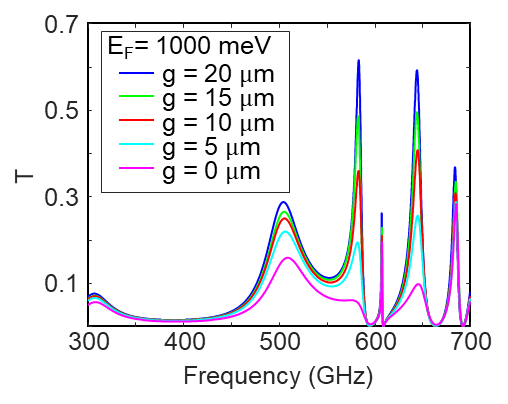 |
| (c) | (d) |
| Figure S5. Simulation of active modulation. (a) 3-D model of the unit with graphene and ion gel in periodic boundary. (b) Transmittance modulation with various chemical potentials. (c) Transmittance with various gaps for graphene at E_F_=0 eV (d) Transmittance with various gaps for graphene at E_F_=1000 meV. | |

The active modulation of the hybrid state is investigated with finite element method simulation in Ansys HFSS. Figure S5(a) depicts the 3-D model in periodic boundary, where a resistive sheet with dispersive impedance calculated from Kubo formula represents the CVD graphene. The sheet conductivity can be obtained from the intra-band term of the Kubo formula

S-(4)

$$\sigma_{g}\left( \omega,\mu_{c},\Gamma,T \right)=\sigma_{g,intra}+\sigma_{g,inter}\approx\sigma_{g,intra}=\frac{-je^{2}\mu_{c}}{\pi h^{2}\left( \omega-j2\Gamma\right)},$$

where *e* is the electron charge, $\omega$ is the angular frequency, $h$ is the reduced Planck’s constant, $\mu_{c}$ is the chemical potential of graphene, and $\Gamma$ is the phenomenological scattering rate for CVD graphene.^S3^ The simulated modulation of the transmittance is illustrated in Figure S5(b). The hybrid state is modulated from 0.51 to 0.24, and the SPP EOT is modulated from 0.25 to 0.14 with a chemical potential sweeping from 0 to 1 eV. Compared to the data in Figure S4(b), the first order SP EOT shows much stronger attenuation than the hybrid state due to the strongly localized field at the plasma frequency.

Simulation was carried out to optimize the position and shape of the graphene structures on the top surface, as shown in Figure S5(c)(d). According to the simulation result, a larger active modulation range can be acquired with larger graphene areas. However, graphene transferred on silicon substrate has an inevitable fundamental doping level (from air absorption and photoresist residue doping), which will significantly influence the maximum transmittance if graphene covers all the metal hole area. Consequently, etched graphene with g = 5 μm was chosen to perform the active modulation of the hybrid mode.

### Graphene resistance

| 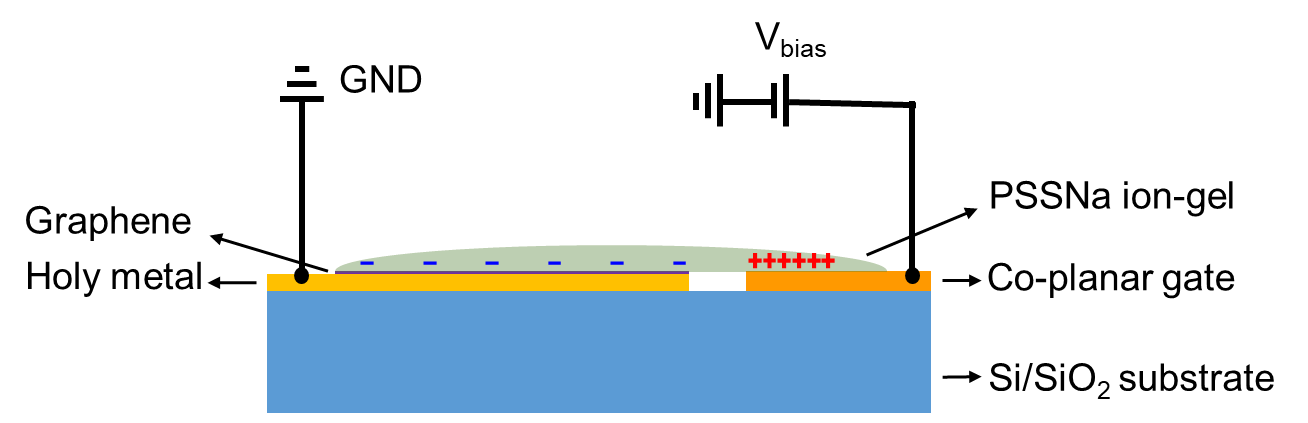 | |
| --- | --- |
| (a) | |
| 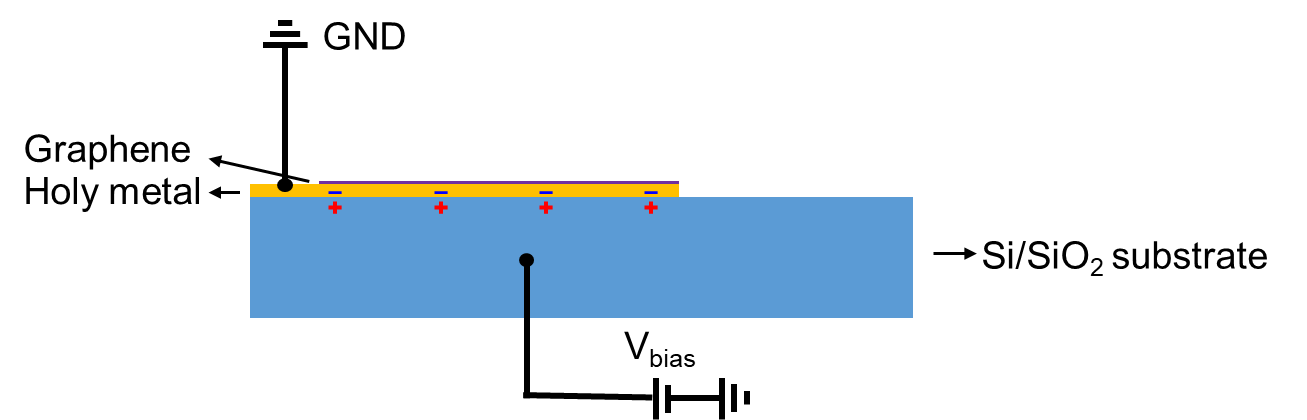 | |
| (b) | |
| 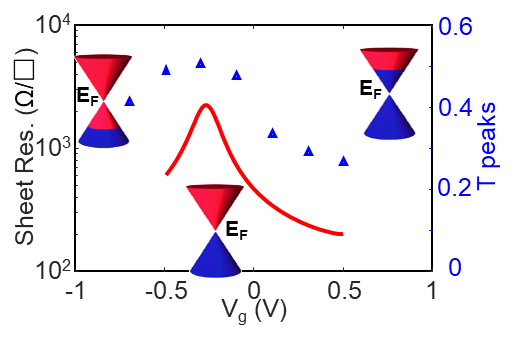 | 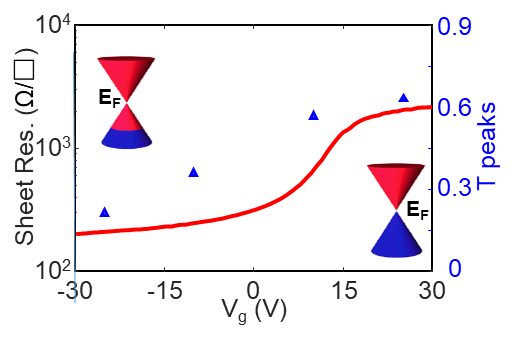 |
| (c) | (d) |
| Figure S6. (a) Biasing set-up configuration for the PSSNa ion-gel gating device. (b) Cross-section illustration of the silicon gating device. (d) Cross-section illustration of the silicon gating device. (c) Resistance-voltage response of a graphene transistor top-gated by PSSNa on the same silicon substrate. The blue triangles represent the peak values of the transmittances at 500 GHz at various bias. (d) Resistance-voltage response of a graphene transistor biased by the same silicon substrate. The blue triangles represent the peak values of the transmittances at 580 GHz at various bias. | |

The electric biasing setup for the two active modulation approaches is illustrated in Figure S6 (a)(b). To reveal the electronic properties and modulation capability of the F-P mode dominant case (loosely coupled hybrid mode), graphene layer was fabricated and gated by solution-processed poly (styrenesulfonic acid sodium salt) (PSSNa) on the same silicon substrate using a co-planar gate. And, silicon gating for carefully patterned graphene was to demonstrate the modulation of the ‘effective SP’ mode dominant case (tightly coupled hybrid mode). The DC current-voltage responses were characterized at room temperature, and the extracted resistance-voltage curve is illustrated in Figure S6 (c)(d). For ion-gel gated structure, the sheet resistance varies from 2300 to 200 Ω/□, revealing a large modulation range over one order. The Dirac point is at -0.3 V, where both the sheet resistance and the transmittance reach peak values. For the silicon gate with 100 nm SiO_2_ has much smaller dielectric capacitance compared with ion-gel coplanar electric double layer capacitances (EDLCs), the Dirac point of silicon-gated graphene shifts to 25 V. Additional reasons for the shift can be attributed to the higher level of the air absorption doping. The silicon gated modulation is mainly for the spoof SPP mode as the graphene is placed on the surface. However, the hybrid mode can exist below the effective plasma frequency ($f_{eff}$), which can be significantly modulated at 500 GHz by using graphene with respect to the spoof SPP mode at 583 GHz, as shown in Fig. 3d.

### Comparison table

The comparison of this work and other active metamaterials modulated by using graphene is shown in Table I. Notably, the applied bias of this work could be the lowest, and the modulation range of this work could be the best, which is even higher than the ideal simulation data.

TABLE I

Comparison of Active Metamaterials Modulated with Graphene

| **Ref.** | **Frequency** | **Structure** | **Graphene shape** | **Bias range** | **Absolute T/R range** |  |  |  |  |  |
| --- | --- | --- | --- | --- | --- | --- | --- | --- | --- | --- |
| 18 | 1.5~3 THz | SP EOT | Graphene ring | none | <30% (Simulated T) |  |  |  |  |  |
| 19 | 35~45 THz | SP EOT | Graphene strip | 0~150 V | <4% (T) |  |  |  |  |  |
| 22 | 0.5~2 THz | Metamaterials | Graphene layer | 0~600 V | <13% (T) |  |  |  |  |  |
| 23 | 2~4 THz | Graphene Plasmonics | Graphene strip | -2.2~-0.5 V | <12% (T) |  |  |  |  |  |
| S4 | 150~300 THz | F-P cavity | Graphene layer | -3~1 V | <7% (R) |  |  |  |  |  |
| S5 | 0.4~0.6 THz | EOT with reflector | Graphene layer | -2~2 V | <22% (R) |  |  |  |  |  |
| S6 | 8~12 GHz | SP EOT | Graphene layer | 0~4 V | <23% (Simulated T) |  |  |  |  |  |
| S7 | 0.3~0.6 THz | SP EOT | Graphene layer | -20~20 V | <25% (T) |  |  |  |  |  |
| S8 | 2.2~2.9 THz | Chiral metamaterials | Graphene layer | 0~5 V | <10% (T) |  |  |  |  |  |
| **This Work** | 0.4~0.6 THz | Hybrid state | Graphene layer | -0.3~0.5 V  -25-25 V | <25% (T)  <38% (T) |  |  |  |  |  |

T/R denotes transmittance/reflectance.

### References

1. Garcia-Vidal, F. J. et al. Light passing through subwavelength apertures. *Rev. Mod. Phys.* 82, 729-787 (2010).
2. Ju, L. et al. Graphene plasmonics for tunable terahertz metamaterials. *Nature Nanotech.* 6, 630-634 (2011).
3. Othman, M. A. K. et al. Graphene-based tunable hyperbolic metamaterials and enhanced near-field absorption. *Optics Express*, 21(6), 7614 (2013).
4. J. Rodriguez, F. et al. Solid-State Electrolyte-Gated Graphene in Optical Modulators. *Adv. Mater.* 1606372 (2017).
5. Chen, X. et al. Electrically Tunable Perfect Terahertz Absorber Based on a Graphene Salisbury Screen Hybrid Metasurface. *Adv. Optical Mater.* 1900660 (2019).
6. Nan, J. et al. Actively modulated propagation of electromagnetic wave in hybrid metasurfaces containing graphene. *EPJ Appl. Metamat.* 7, 9 (2020).
7. Gao, W. et al. High-contrast terahertz wave modulation by gated graphene enhanced by extraordinary transmission through ring apertures. *Nano Lett.* 14, 1242-1248 (2014).
8. Kim, T.T. et al. Electrical access to critical coupling of circularly polarized waves in graphene chiral metamaterials. *Sci. Adv.* 3, e1701377 (2017).
